# The Neonatal and Juvenile Pig in Pediatric Drug Discovery and Development

**DOI:** 10.3390/pharmaceutics13010044

**Published:** 2020-12-30

**Authors:** Miriam Ayuso, Laura Buyssens, Marina Stroe, Allan Valenzuela, Karel Allegaert, Anne Smits, Pieter Annaert, Antonius Mulder, Sebastien Carpentier, Chris Van Ginneken, Steven Van Cruchten

**Affiliations:** 1 Comparative Perinatal Development, Department of Veterinary Sciences, University of Antwerp, 2610 Wilrijk, Belgium; Laura.Buyssens@uantwerpen.be (L.B.); Marina.Stroe@uantwerpen.be (M.S.); Allan.Valenzuela@uantwerpen.be (A.V.); Chris.VanGinneken@uantwerpen.be (C.V.G.); 2 Department of Pharmaceutical and Pharmacological Sciences, KU Leuven, 3000 Leuven, Belgium; Karel.Allegaert@uzleuven.be (K.A.); Pieter.Annaert@kuleuven.be (P.A.); 3 Department of Development and Regeneration, KU Leuven, 3000 Leuven, Belgium; Anne.Smits@uzleuven.be; 4 Department of Hospital Pharmacy, Erasmus MC Rotterdam, 3000 CA Rotterdam, The Netherlands; 5 Neonatal Intensive Care Unit, University Hospitals UZ Leuven, 3000 Leuven, Belgium; 6 Department of Neonatology, University Hospital Antwerp, 2650 Edegem, Belgium; Antonius.Mulder@uza.be; 7 Laboratory of Experimental Medicine and Pediatrics, University of Antwerp, 2610 Wilrijk, Belgium; 8 Facility for SYstems BIOlogy Mass Spectrometry, KULeuven, 3000 Leuven, Belgium; Sebastien.Carpentier@kuleuven.be

**Keywords:** pediatric pharmacology, juvenile pig model, translational research, drug discovery, drug development, drug safety, PBPK

## Abstract

Pharmacotherapy in pediatric patients is challenging in view of the maturation of organ systems and processes that affect pharmacokinetics and pharmacodynamics. Especially for the youngest age groups and for pediatric-only indications, neonatal and juvenile animal models can be useful to assess drug safety and to better understand the mechanisms of diseases or conditions. In this respect, the use of neonatal and juvenile pigs in the field of pediatric drug discovery and development is promising, although still limited at this point. This review summarizes the comparative postnatal development of pigs and humans and discusses the advantages of the juvenile pig in view of developmental pharmacology, pediatric diseases, drug discovery and drug safety testing. Furthermore, limitations and unexplored aspects of this large animal model are covered. At this point in time, the potential of the neonatal and juvenile pig as nonclinical safety models for pediatric drug development is underexplored.

## 1. Introduction

Clinical pharmacology aims to evaluate and understand drug-specific (side)-effects based on pharmacokinetics (PK) and pharmacodynamics (PD). Pharmacokinetics (absorption, distribution, metabolism and excretion; ADME) describes drug concentration time courses in a given compartment, like blood, cerebrospinal fluid or subcutaneous tissues. When considering the systemic circulation, the concentration time course is dictated by volume of distribution (Vd) and clearance (CL) as primary PK parameters. Pharmacodynamics describes the link between drug concentrations (in the systemic circulation and/or at the site of action) and effects or side-effects over time. Throughout pediatric life, it is reasonable to anticipate that both PK as well as PD are affected by developmental changes, reflecting both maturation and growth or weight/size changes [1].

All ADME processes display maturational changes, so that extrapolation and dosing based on simple linear (e.g.,/kg or/BSA) or allometric scaling (kg^x^) commonly results in over- or under-exposure in specific subpopulations [2]. Besides the physicochemical properties of a given drug, the rate (and extent) of absorption (typically represented by an absorption rate constant) is affected by developmental physiology, i.e., gastric emptying time, pH or gastric fluid volume; intestinal transit time, bile concentrations or volumes; first pass effect factors like drug-metabolizing enzymes or drug transporters [3]. As an illustration, the oral bioavailability of midazolam is highest in preterm neonates (49–92%), 25–85% in infants and 21–30% after infancy, mirroring ontogeny of the activity of enzymes involved in first pass metabolism [4]. The same holds true for maturational patterns on distribution. After reaching the systemic circulation, a drug will distribute to different compartments (tissues, organs). This distribution behavior is determined by the physicochemical properties of the drug (size, degree of ionization at physiological pH, lipophilicity or water solubility), but further depends on maturational changes such as cardiac output and organ-specific blood flow, and body and plasma composition, as well as the interaction between them, e.g., plasma and tissue protein binding kinetics. To illustrate this, the body water/kg body weight proportion is up to 80–90% in preterm neonates, with a subsequent decrease to 60% after infancy [5]. Protein binding capacity is dependent on maturational plasma protein binding capacity, as illustrated for, e.g., vancomycin [6]. The liver, followed by the small intestine and the kidneys, are the major sites of drug metabolism. In the liver, changes in activity for both phase I and II enzymes are observed in an iso-enzyme-specific manner [1,7]. Maturational changes in these organs will be further discussed later in this review. Excretion is mainly by the kidney via glomerular filtration and tubular secretion and reabsorption, but occasionally also occurs by hepato-biliary or respiratory routes. Similar to hepatic metabolism, each of these processes displays characteristic scenarios of maturation. Compared to glomerular filtration, tubular functions mature more slowly, with tubular secretion at adult equivalent level by 15 months, and tubular reabsorption by 24 months [8,9]. Maturational aminoglycoside clearance closely follows the development of glomerular filtration, while digoxin clearance is, in part, affected by the maturation of tubular secretion capacity [10,11]. These PK profiles are further affected by a diverse set of nonmaturational covariates, including but not limited to genetics, disease characteristics (e.g., obesity, chronic inflammation, critical illness, chronic kidney disease, asphyxia) or environmental factors (e.g., type of nutrition, comedication, drug formulation, treatment modalities like therapeutic hypothermia) [12].

In pediatric pharmacotherapy, it should not be taken for granted that a given level of drug exposure to adults will result in similar drug effects in children, as concentration-effect profiles may also display developmental PD [13]. This refers to the ontogeny of biologic systems, and how drug (side-) effects are determined by developmental stage. Safety PD outcome parameters of specific interest relate to neurodevelopmental outcome, growth (length, specific organs) or pubertal development. Differences in chloride flow direction following activation of the GABA receptor, the maturational effect of cyclosporine on monocyte proliferation or the maturational QTc prolongation serve as illustrations of the critical relevance of maturational PD [14,15,16].

It is critically important that all sources of information are leveraged to optimize dose selection for neonates, infants or children, taking their (patho)physiology and associated variability into account and covering both PK and PD. Such sources include data from previous studies in humans (adults, other pediatric subpopulations), but also nonclinical juvenile animal models, in vitro systems, and in silico models. Depending on the drug development program, each of these methodological dimensions can be used to varying degrees, considering its strengths and limitations.

Among nonclinical in vivo models, large animals, like the neonatal and juvenile pig, are of increasing interest in pediatric drug development from two perspectives. First, to investigate and consequently better understand the mechanism of a disease, particularly when it is unique to pediatric patients. Second, the model may also provide important safety data for the pediatric population when performing juvenile toxicity studies. The choice of species and the design of juvenile toxicity studies are therefore the result of a series of complex considerations, including the therapeutic use of the drug, the age at which children will be treated, the duration of treatment, and potential age- or species-specific differences in efficacy, PK, or toxicity observed in adult animals [17]. The utility of a ‘leverage concept’ for dose determination and drug development programs in neonates has recently been described in this journal [18]. The following scenarios can be distinguished:

Pediatric disease similar to that in adults and/or older pediatric patients where dosing is known for adult and/or older pediatric patients = extrapolation of efficacy from adults to pediatric patients is permitted, and even supported [19,20].

Pediatric disease related but not similar to that in adults and/or older pediatric patients where dosing is known for adult and/or older pediatric patients = additional information can be leveraged from either in vitro or in vivo models to guide initial dosing [19,20].

Pediatric disease unique to a given (sub)population within pediatrics, where these drugs are not utilized for these specific diseases in adults [19,20].

Even in the setting of similarity, additional research in juvenile animals may still be warranted when concerns related to developmental toxicology (like growth, neurodevelopment, kidney or cardiovascular system) should be addressed. Only about 10% of the 400 products (almost exclusive new drug approvals) of which the labels were reviewed between 1998–2009 by the FDA contained information on juvenile animals [21]. In a recent survey on European Pediatric Investigation Plan (PIP) decisions (2007–2017, 229 drugs) with juvenile animal requests, general toxicological studies were the most applicable study designs, with infectious diseases, endocrinology, neurology and cardiovascular diseases being the most common therapeutic areas. As anticipated, about 80% of these studies were in rats, while studies in pigs were limited (4.2%) [22]. Interestingly, a recent European Medicines Agency (EMA) analysis on juvenile animal studies in the field of anticancer drug research documented that juvenile models also generated evidence regarding new target organ toxicity (kidney, central and peripheral nervous system, impaired learning or memory, cardiac system) or increased severity of toxicity (including mortality rate) [23]. At the other end of the spectrum, with diseases that are unique to a given subpopulation within pediatrics, pig models can be instrumental in drug discovery and development for accurate mechanistic understanding of the disease or condition. Specific to neonates, this has been described for, e.g., necrotizing enterocolitis (NEC), resuscitation practices, or asphyxia. Studies in pigs have established the essential roles of prematurity, microbial colonization and enteral nutrition in the pathogenesis of NEC [24]. The (juvenile) pig is also an important animal model in research on human resuscitation [25]. In addition, in vivo data generated in neonatal animals—including (mini)pig—facilitate the development of a neonatal physiology-based PK (PBPK) model during therapeutic hypothermia [26].

The main goal of this review is to provide an overview of the current knowledge of similarities between pigs and humans in view of pediatric pharmacotherapy, including different fields of application in which the neonatal or juvenile pig has proven to be useful from either a safety or mechanistic point of view. Furthermore, the limitations of the model and gaps in current knowledge are discussed. 

## 2. Anatomical, Physiological and Developmental Similarities between Pigs and Humans

### 2.1. Characterization of the Pig As a Relevant Animal Model 

From a drug discovery and development point of view, both mechanistic understanding and safety are key aspects in the process. Animal models used in the drug discovery process usually share pathophysiological traits with humans, which facilitates the identification of molecular targets and PD parameters. On the other hand, similar ADME process are desirable when the model is to be used in safety studies. Among nonrodent species, the pig presents several advantages related to similarities in anatomy and physiology when compared to humans that have been extensively reported and reviewed in literature [27,28,29,30,31]. Besides species similarity, the large litter size allows for a reduction in animals to be kept for breeding purposes, further facilitated by the short reproductive cycle [32]. Moreover, this enables researchers to place siblings into different experimental groups. Finally, large litter sizes usually lead to the spontaneous birth of intrauterine growth restricted (IUGR) pigs [33], which will be discussed later in this review. Pigs, especially minipigs (for size reasons), are relatively easy to handle and train [34,35], and their larger size at birth when compared to dogs or nonhuman primates (NHP) facilitates sampling at early stages. This larger size at birth, more similar to human neonates, facilitates the adaptation of NICU equipment for its use in pigs, increasing their translational value. Moreover, all routes of administration are possible and they represent the best model for dermal studies [36]. Pigs are precocial animals, which allows early separation from the mother. This can be beneficial in studies that require artificial rearing. Some disadvantages that will be further discussed in Section 4 include the more limited historical control data available for (mini)pigs than dogs and NHPs at present, the different placentation and a relatively more mature respiratory and musculoskeletal systems compared to human newborns. This is nevertheless compensated for by the easy access to spontaneous IUGR and preterm pigs showing different degrees of immaturity in several organs (see Section 3.2.2). These features have made the pig a well-accepted translational model, and in recent decades, several small to very small pig breeds have been developed specifically for laboratory use. For a detailed overview of the development and use of miniature, micro- and mini- pigs in biomedical research, we refer to Swindle et al., 2012 [29]. Within Europe, one of the most commonly used, purpose-bred pigs is the Göttingen Minipig. It is the result of crossbreeding the Minnesota Minipig, having a small stature and a gentle temperament, the Vietnamese potbelly pig with low body weight and high fertility, and the German Landrace pig for its white skin. Due to the fact that it is a genetically coherent breed that is easy to handle and can be housed in facilities originally designed for Beagle dogs, it is a popular model for drug development programs. Besides purpose-bred pigs, the wide variety of breeds used for pork production provides researchers with a broad spectrum of swine varieties, with different genetic backgrounds, sizes and fattening levels. 

Although pigs have been models in biomedical research for decades, efforts are still being made to better characterize this model. As an example, the COST action called SALAAM (Sharing Advances on Large Animal Models), a EU-funded research network (2014–2018), connected researchers from 24 European countries with the objective of improving large animal models and phenotyping protocols, developing selection criteria for animal models and creating and sharing data and samples to advance the use of large animals (pigs, small ruminants and rabbits) where they may be of interest. However, despite already having in-depth knowledge on the anatomy and physiology of the (mini)pig and attempts to further characterize ADME processes in minipigs for their use in pharmaceutical research [32,37,38,39], more work is needed, especially in neonatal and juvenile pigs.

### 2.2. The Pig in Pediatric Research

#### 2.2.1. Pig and Human Postnatal Development

In order to assess the feasibility of the neonatal and juvenile pig as models for pediatric drug development, an in-depth characterization of this model must be carried out in the first place, followed by a comparison with the anatomical, physiological and ADME characteristics in the corresponding pediatric age groups. Our group has already reported on the age-related maturation of organ weights in the developing Göttingen Minipig in an effort to further develop a PBPK model [35], but more data are needed. The implementation of this model would benefit from data on microsomal protein per gram of liver and abundance on drug metabolizing enzymes during development, or from a better understanding of pig orthologues for human cytochrome P450 (CYP) enzymes. For the developing domestic pig, the anatomy, physiology and the absorption, distribution and excretion of drugs have been reviewed by Gasthuys et al. [40]. As some of the above data are publicly accessible in the ICH S11 guidelines on nonclinical safety testing in support of the development of pediatric pharmaceuticals [36], we will only highlight some key points in this review. The EMA has established different age categories within the pediatric population [41], and many similarities between human and Göttingen Minipig organ development (as the reference breed used in the pharmaceutical industry [42] were reported in the ICH S11 guidelines [36]. 

**Figure 1:**
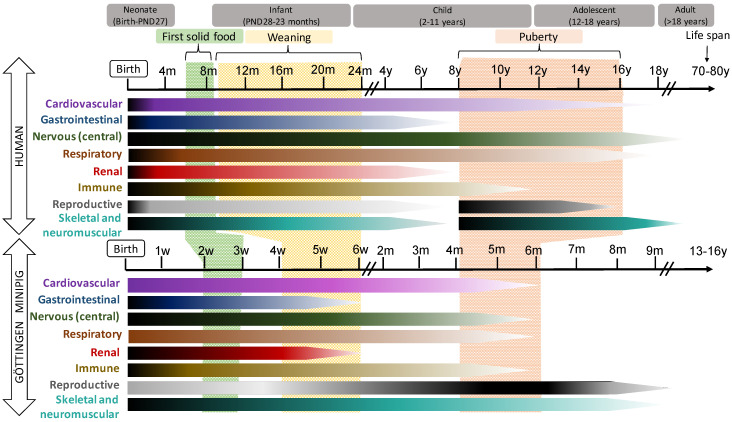
Schematic representation of the postnatal development of different organ systems in human (top) and Göttingen Minipig. In the horizontal bars, the intensity of the maturation process is represented by dark (more intense) and light (less intense) tones. The time bar represents weeks (w), months (m) or year (y) of life.

In general, pigs and humans share many developmental milestones: The patterns of development of the gastrointestinal tract (GIT), the cardiovascular, the CNS systems and the eye are quite similar in both species, while renal, immune and reproductive development occur slightly earlier and more quickly in humans than in pigs. These data are illustrated in Figure 1. 

Regarding the GIT, its importance lies in its key role in drug absorption, as the first step of ADME. It is also one of the systems, together with the respiratory system, that causes more referrals to the NICU, and is therefore of great interest in pediatric medicine and pharmacology. Although some anatomical differences exist, the physiology of digestion is approximately the same in juvenile pigs and humans. As previously mentioned, the developmental pattern is also similar in pigs and humans, as depicted in Table 1. As a component of the GIT, the liver serves a central role in digestion, but also in the metabolism of xenobiotics, including drugs. Our group has been working on the characterization of (hepatic) drug metabolism for several years now, with a focus on the Göttingen Minipig [43,44,45]. In contrast to the other ADME processes, there is, as yet, no comprehensive review that covers (hepatic) drug metabolism in (mini)pig; therefore, we will devote the next section of this review to this important disposition process. Regarding the cardiovascular system, pig heart is the most similar to human heart among domestic species, although the position is slightly different due to body orientation [46]. Other differences are shown in Table 1. In spite of minor dissimilarities, the pig is considered a sound model for cardiovascular medicine, surgery and even cardiac transplantation. The cardiovascular system is involved in drug distribution, but it is the concentration of serum proteins, rather than blood flow (Table 1), that can have a significant impact on that aspect. Drug distribution is also determined by tissue composition, mostly the water/fat ratio. As described for plasma proteins (Table 1), pig and human water/fat ratios differ in the earliest stages of development (with humans having a larger amount of fat) but are comparable when reaching infancy ([40]). The (perinatal) development of the CNS in pigs is very similar to that in humans, and is therefore considered a solid model [47]. Regarding the renal system, although the external anatomy and some functional parameters are fairly similar, the pig’s nephrogenesis is different from that in human (Table 1). This should be considered, especially when assessing drugs with a predominant renal excretion pathway. The development of the immune system is different in pigs and humans, starting with the lack of passive immunity at birth in the case of the former. On the other hand, the high level of similarity between the pig and human immune system (Table 1) translates into good acceptance of the pig model [31,48,49]. In fact, the lack of immunoglobulin transfer—together with the precocial development of the pig—can be an advantage for studies on immune system development, since it is possible to control environmental factors (i.e., the mother) that have been shown to impact postnatal development of adaptive immunity [48]. The differences in the development of the reproductive system are marked by a more active period in neonates and young infants in the case of the human, while only minor changes are observed in pigs before puberty (4 to 5 and 5 to 6 months of age in female and male minipigs, respectively). However, the impact of sex is manifest, even in early life stages, affecting the development of the CNS [50], the immune system, the lungs [51] and even metabolism [52] and survival rates [53] in pigs and humans. 

Due to the precocial nature of pig neonates, the skeletal and neuromuscular systems are more developed at birth. This is obviously different in human beings, making comparison between the two species difficult. This could, however, be an advantage for neurodevelopment studies, since behavioral assessments can be performed from birth onward, in contrast to other models such as rats and dogs [28]. Regarding the respiratory system, there are some maturational differences (Table 1), although respiratory disease models (meconium aspiration and lavage models, further discussed later in this review) have been developed in neonatal pigs [54]. Moreover, it is possible to reach the required degree of immaturity of the respiratory system in a pig model by using preterm or IUGR pigs.

**Table 1:** Summary of main similarities and differences in maturation of different organ systems in pigs and humans.

Organ System	Feature	Similarity	References
Gastrointestinal	Physiology of digestion	Very similar	[55]
	Ontogeny of digestive enzymes	Similar in most cases (more than the rat)	[56]
	Neonatal gastric pH	Different: pigs show adult-like values early in life (5 days of age) and humans reach that point at 2 years of age	[35,57,58]
	Gastric emptying	Maturation of gastric emptying with age has not been established in pigs. Prolonged emptying is expected in newborn pigs, as observed in humans	[56]
	Intestinal transit	Similar: longer in neonates than juvenile/adults	[56,59]
	Intestinal surface	Similar: smaller than juvenile/adults, leads to similar nutrient absorption	[40,60]
	Microbiome	Similar: mainly consists of Firmicutes and Bacteriodetes phyla	[61]
	Liver	Similar relation to body weight in adults (about 2%)	[35]
		Slightly higher ratio in human (around 5%) than minipig (3%) neonates	
Cardiovascular	Drainage	Different	[40]
	Main central vessels	Different relative importance	
	Cardiac output	Different	
	Cardiac myocyte maturation	Similar (compared to other species)	[62]
	Serum proteins (albumin and globulins)	Different in neonatal pigs and humans, but even at infant stages	[40,63]
Central nervous	Anatomical complexity	Similar	[28,35]
	Distribution of grey and white matter	Similar	
	Brain growth pattern	Similar	
Renal	Nephrogenesis	Different: completes after weaning (3 weeks of age) in pig and 34–36 weeks gestational age in humans	[64]
	Glomerular filtration rate	Similar maturation: adult levels at 8 weeks (pig) and 1 year (human) of age	[65]
	Effective renal plasma flow	Within the same range in growing pigs and children	[66]
	Urinary pH		[66]
Immune	Immune genes	High similarity (>80%)	[67,68]
Skeletal and neuromuscular	Development	Different: faster in pigs, in which locomotion patterns reach mature levels as early as 8 h after birth	[69]
Respiratory	Anatomy and histology	Similar	[70]
	Maturation	Faster	[36]
	Alveoli multiplication	Earlier in pigs	[71]

#### 2.2.2. Hepatic Drug Metabolism in the Neonatal and Juvenile Pig

Hepatic Phase I drug metabolism mediated by CYP enzymes has been investigated extensively in adult conventional pig strains [72,73,74,75,76,77,78,79,80,81] and minipig strains [43,44,79,82,83,84,85,86,87] over the past 30 years. Knowledge on the ontogeny of these processes in the neonatal and juvenile population is much more limited. Particularly in neonates, it is crucial to predict drug disposition correctly in order to avoid inefficacy due to underdosing or adverse effects caused by overdosing. Recently, CYP activity was determined in the neonatal and juvenile conventional pig [88] and the Göttingen Minipig [45] in different age groups using several human CYP450 substrates. As such, substrate specificity was examined and CYP450 activity levels in (mini)pig were compared to those in human. In the Göttingen Minipig, we found that CYP450 enzyme activity increased postnatally. However, differences in onset and speed in development were observed: CYP1A2- and CYP2D6-like activity levels increased fast during the first week of life, whereas CYP2C9- and CYP3A4-like activities matured more slowly, reaching their highest levels in 1-month-old pigs [45], corresponding roughly to a 2-year-old child [89,90,91,92,93]. In the conventional pig, similar results were obtained [88]. In addition, no sex-related differences were observed in the neonatal and juvenile age groups regarding the CYP450 ontogeny patterns until puberty [45,88]. With regard to CYP450 protein abundance, some research has already been conducted on the conventional pig [88], and this question is currently being addressed in the Göttingen Minipig by our group. In general, activity and abundance data correlate well, although CYP isoform-specific differences have been reported [88]. Regarding Phase 2 metabolism, data are scarce. A recent in vitro study on 1-day and 2-, 5-, 10- and 20-week old male Camborough-29 pigs showed that UDP Glucuronosyltransferase (UGT) enzyme activity increased from day 1 until week 10, followed by a decline at around week 20 [94]. An in vivo study with ibuprofen in 1-, 4-, and 8-week old, and 6–7-month old, mixed breed pigs also showed UGT activity already in neonatal pigs [95]. In our group, UGT activity was investigated in Göttingen Minipigs with age groups ranging from the late fetal stage until postnatal day 28, and adults [45]. From PND 7 onward, UGT activity increased without sex-related differences [45]. In the youngest age groups (gestational day age (GDA) 84–86, GDA 108, postnatal (PND) day 1 and 3), activities were below the lower detection limit when using a luminescence-based assay [45]. However, immunohistochemical analysis showed that even in the late fetal stages, UGT1A could be detected. In accordance with the activity results, UGT1A detection increased with age [45]. In general, it can be concluded that UGT enzymes are expressed from an early age, but further characterization of the different isoforms is needed in order to better predict drug disposition in this animal model. With regard to drug transport (often referred to as Phase 0 for uptake transporters and Phase III for efflux transporters), the data in the pig are even more scarce. For the Göttingen Minipig, we performed a semiquantitative assessment of P-glycoprotein (P-gp, encoded by the Multidrug Resistance Gene, *MDR*) in the liver of neonatal and juvenile pigs and fetuses using immunohistochemistry. No difference was observed in P-gp expression between livers from GDA84 to adult animals (1.5–3 years of age) [43]. 

When comparing the above data with the ontogeny profile of the drug disposition processes in human, which have been reviewed extensively elsewhere [96,97,98], remarkable similarities are present. For UGT and P-gp, the ontogeny profile on protein and activity level, if assessed, is very similar. With regard to the CYP activity, the interpretation is more complex. The slow maturation profile of CYP2C9 and CYP3A4 activity in (mini)pigs corresponds well with the pediatric population. For CYP1A2 and CYP2D6, there appears to be an earlier onset of activity in the pig than in human, and CYP2D6 activity in general appears to be much higher than in human. Still, when comparing the pig with man, one needs to be very cautious, as studies may use different substrates or other testing conditions, which may confound the results and, as such, species comparisons. This said, even when not directly translatable to human, in vitro and in vivo drug metabolism data in juvenile animals are critical, as they may explain differences in efficacy or toxicity with the human population, and they can be used in mathematical models to better predict exposure, especially in the very young age groups, as further discussed in the next section. 

#### 2.2.3. PBPK Models in the Neonatal and Juvenile Pig

In general, PBPK models provide a mechanistic framework for predicting drug exposure in special populations via a ‘full bottom-up’ modelling strategy. Input data include: (i) drug-specific data, including physicochemical properties and in vitro disposition data (e.g., metabolic rates, plasma protein binding and transepithelial permeability; and (ii) quantitative mapping of the physiology of the biologic system of interest (e.g., neonatal human or pig). The fact that predictions of in vivo drug disposition can be made based on ‘first principles’, i.e., with limited need for in vivo animal or clinical data remains one of the unique strengths of PBPK models. However, at present, this important advantage also comes at a cost, i.e., (i) predicting population variability in drug disposition processes remains challenging; (ii) initial evaluation of the predictive performance is difficult in populations (e.g., neonates) where limited clinical data have been collected, especially for first-in-class compounds. 

When no or limited clinical data are available for model verification, building a PBPK model for a corresponding animal model carries the potential to indirectly inform (and improve) the desired PBPK model in the human target population. This strategy has, for instance, been applied to determine an adequate IV dosing regimen for oseltamivir in neonates and infants. In this case, the availability of plasma and liver PK data obtained after oral and IV administration in newborn and adult marmosets was instrumental in establishing and verifying neonate/adult PBPK models for oseltamivir in this species. Moreover, confidence in the ultimate human neonatal PBPK models was achieved by input from both the newborn marmoset PBPK model (in terms of immaturity) and from the adult human PBPK model (more in terms of incorporation of drug-specific data). 

It is anticipated that also in (neonatal and juvenile) pigs, PBPK models will be increasingly used to enhance the chance for successful development of PBPK models in equivalent human populations. Consistently, best practice guidelines for building PBPK models for ‘novel’ species (such as minipig) have very recently been proposed [99]. Specifically, for supporting the development of (mini)pig PBPK models, it is critical that the relevant physiological descriptors are available in sufficient detail. For the adult Göttingen Minipig, Suenderhauf and Parrott [39] were the first to publish a compilation of gastrointestinal pH values and transit times, along with organ sizes and blood perfusion rates. The Advanced Compartmental and Transit (ACAT) concept was used to describe the drug absorption processes. The PBPK model implementation of these physiological data was verified with moxifloxaxin and griseofulvin, both after IV and oral administration. In a follow-up study [100], the same research group explored gastric emptying times (GET) in minipigs using paracetamol as a model drug. Their findings demonstrated high variability in GET values that also turned out to be higher as compared to humans. Another absorption-related application of PBPK modelling included the development of modified release formulation for a compound with region-dependent absorption [101]. Furthermore, using both slowly eliminated and rapidly cleared model compounds, the utility of PBPK modelling to extrapolate PK profiles in minipigs to the human situation has been illustrated [102]. More recently, predictions of the volume of distribution in minipigs (Vd,ss) based on tissue compositions (e.g., relative amounts of neutral lipids, phospholipids and intra/extracellular water) have been implemented, thereby making a major step in the further refinement of a Göttingen Minipig PBPK concept [103]. To support establishing minipig PBPK models for the younger age groups, we previously reported organ weights and GI pH values of Göttingen Minipigs between the fetal stage and 5 months of age as a first step [35]. Several critical data, such as liver blood flow, abundance and activity of drug transporters, scaling factors, etc. are still missing, but with the increasing availability of ontogeny data for both phase I and phase II biotransformation pathways, the pieces of the puzzle to build a neonatal Göttingen Minipig PBPK model are falling into place.

Ultimately, development of PBPK models for human neonates with a specific disease is also expected to benefit from the learnings obtained when developing PBPK models in the corresponding neonatal animal model. As recently proposed by our group [26], the goal to establish both a Göttingen Minipig and human neonatal PBPK model is expected to prove uniquely instrumental in predicting the influence of therapeutic hypothermia on PK of key drugs used in asphyxiated neonates. The advantage of incorporating a PBPK platform in such an endeavour lays in the possibility to deconvolute the distinct influences of disease severity, therapeutic options and maturational physiology on PK. Importantly, this latter project will also require tailored in vitro studies—for instance, to study the influence of temperature on intrinsic enzyme-mediated clearance—to be used as input data for the PBPK models. For the purpose of this project, the proposed workflow for the development of PBPK models will be as follows: (i) build healthy neonatal pig PBPK model; (ii) modify the PBPK model to reflect hypotheses regarding the impact of asphyxia and/or hypothermia on physiology and PK pathways; (iii) iterative evaluation and improvement of PBPK-based predictions against PK observations obtained in neonatal pigs; (iv) establish a healthy neonatal human PBPK model; (v) build a human neonatal PBPK model for asphyxiated neonates, implementing the verified hypotheses regarding disease impact as evaluated in the corresponding Göttingen Minipig disease model.

## 3. Fields of Application

### 3.1. History of Pharmacological Studies in Pigs

Some of the earliest works including the pig (or its tissues) as a model for human pharmacology go back to the early sixties, when the effects of chlorpromazine on the activity of pig plasma cholinesterase [104] or the PK of oxazepam in pig were assessed [105]. Around 10 years later, the Canadian Federal Food and Dug Directorate started a series of works to evaluate the absorption of several drugs such as phenylbutazone, sulfisoxazole and sulfadiazine in pigs in order to evaluate their potential use in PK and toxicology studies [106]. In the same decade, more research characterizing the response of pigs (among other species) to different pharmacological molecules was published [107,108,109,110,111]. Also, a review on the use of animal models to assess bioavailability was published considering the pig, together with the dog and the monkey, as a useful secondary standard (rodents are first line) for routine quality control of some drugs [106]. Regarding the use of neonatal/juvenile pigs, the first references appear a bit later. At the end of the seventies, one of the first studies on neonatal pigs evaluated the action of diuretics in the newborn pig in order to qualify it as a model for immature mammals, assess the effect of age and compare the results to those observed in adult animals [112]. Another study, aimed to characterize the tracheal muscle response to different drugs, reported differences with results observed in dogs and highlighted the importance of studies on age-related changes to understand the maturation and development of pharmacological receptors in the airways [113].

In the next 20 years, the awareness of the importance of development on drug disposition rose among pharmacologists, leading to a new research field: neonatal PK [114,115,116]. Since then, the efforts to better understand drug metabolism and safety in pediatric populations have augmented and so has the use of animal, in vitro and in silico models for this sensitive age group. Especially after the implementation of PIPs and Pediatric Study Plans (PSPs) by the EMA and FDA agencies, respectively, the number of studies in neonates and juvenile animals from different species, including the pig [117,118,119,120], has raised substantially. Current advances and research on pharmacology in the juvenile/neonatal pig are discussed below.

### 3.2. Current Pharmacological Research in Pigs

The need for more and better research in pediatric pharmacotherapy and the regulation of authorizations for pediatric drugs have noticeably increased the amount of pediatric nonclinical testing. In this section, we present the most recent advances on pediatric research in pigs as well as ongoing investigations in our group.

#### 3.2.1. Nonclinical Safety Models for Small and Large Molecules

Small molecule drugs have traditionally dominated the pharmaceutical panorama, accounting for 67% of all new drugs approved between 1999 and 2008 [121]. They include any compound with a low molecular weight (less than ~1000 g/mol) manufactured by chemical synthesis. Large molecules or biopharmaceuticals such as (monoclonal) antibodies, recombinant proteins and gene and cell therapies comprise a wide field with respect to the structure, PK and function of these compounds. Even though they represent still a smaller percentage of new drugs approved, the field is rapidly advancing. The pig can potentially be used in nonclinical studies for both types of molecules aiming at pediatric use.

An extensive survey of 24 pharma companies on the use of juvenile animal models in nonclinical trials (1999 to 2008) showed that only 2 out of the 241 drugs included in the survey were tested in minipigs, both of them with dermal preparations for Impetigo and Eczema. In general, findings of this survey depict results that are comparable to adult data for most of the drugs included, novel toxicity in 16 studies and greater exposure (leading to potential toxicity) in about 35% of the cases. More recently, statistics presented by the EMA on PIPs from 2008 to 2016 revealed that 26% of the PIPs contain juvenile animal studies (mostly rat) and none of them used pigs [122]. Rats are the most commonly used species in juvenile animal studies as they show several advantages, of which some are described below (for a more complete list of (dis)advantages, see [36]). Rats are in most cases used in earlier repeated dose and developmental toxicity studies during drug development. This facilitates comparisons of toxicity and systemic exposure between juvenile and adult animals. As this is a widely-used species, extensive historical control data are available. They have a large litter size, pups can be easily fostered, they require a small amount of test compound, they are easily housed, transported and managed. However, there are also several disadvantages to this model species. Several organ systems are less developed at birth compared to man, e.g., ADME characteristics often relate poorly to humans preweaning due to the immaturity of the gastrointestinal tract; blood sampling is often a terminal procedure, especially for the youngest age groups; they are usually not suitable for biopharmaceuticals; and their maturation is much faster than that of humans, which makes the detection of distinct windows of vulnerability difficult. The dog was often used in earlier PIPs, especially when a rodent and nonrodent species were required, but nowadays, often only one nonclinical species, usually the rat, is requested by regulatory authorities, and as such, the number of juvenile dog studies has decreased. Its advantages and disadvantages are also extensively discussed in the ICH S11 guidelines [36]. In brief, the juvenile dog also has the advantage of comparability with adult animals, as this species is often used in adult repeated dose toxicity studies. Pups are relatively large at birth, can be easily handled and postnatal development of most organ systems is relatively similar to that of human infants. However, pups cannot be fostered, they require a larger amount of test compound than rat pups, dogs are seasonal breeders and litter size varies, they are prone to vomiting and drug metabolizing capacity varies with humans. Historical control data are also much more limited compared with rats. Nonhuman primates, and especially cynomolgus and rhesus monkeys, are often considered to be the best representative model for humans, but although they show many similarities with man, they also have some disadvantages as nonclinical model [36]. Nonhuman primates have a protracted development, which makes studying all developmental stages impossible, and they only have single offspring. Additionally, neonatal NHPs are precocious for some organs compared to humans, e.g., musculoskeletal, respiratory, endocrine and CNS system. With regard to the pig/Göttingen Minipig, most advantages have been addressed before (see Section 2). Disadvantages are limited and will be discussed in Section 4. Despite the fact that the piglet appears to be a good translational model for pediatric drug development, only two PIPs including neonatal (2-iminobiotin) and juvenile (concentrate of proteolytic enzymes in bromelain, NexoBrid) minipigs in the nonclinical studies were processed after 2016. Despite the limited use of juvenile pig testing in PIPs, their potential translational value and their advantages over other models are translated into several independent studies collecting information and improving the knowledge on the applicability of the pig in pediatric drug research. For example, among small molecules, studies have been published in recent years addressing the PK of antibiotics, anesthetic drugs and NSAIDs in juvenile pigs. Rifampin was selected as a model compound to provide preliminary proof-of-concept results supporting the use of the juvenile porcine model for nonclinical pediatric PK testing. The pig showed higher similarities in ontogenic changes in PK than other species, including the dog [123]. The distribution and PK of another antibiotic, cefpodoxime, was successfully studied, in this case focusing on cerebrospinal fluid, in juvenile (10–20 days of age) pigs. The neonatal pig model has been widely used in the field of anesthetic drugs to study different aspects from PK and blood-barrier crossing [124] to hemodynamic changes after halothane administration [125] or side effects derived from anesthesia. Some of these effects include neurotoxicity observed in immature patients after anesthesia with propofol, where a pig model was used to identify the underlying mechanisms [126]. In fact, a pig model for the study of anesthetic-induced developmental neurotoxicity has recently been developed [127]. As another example of the usefulness of juvenile pig models in drug development, a study on developmental PK and safety of ibuprofen was published in 2019. Authors demonstrated an effect of age on several parameters including clearance and volume of distribution that is consistent with reports in children, although data are limited and somehow inconsistent. Thus, the pig would be a good model for PK of ibuprofen and potentially other NSAIDs in the pediatric population [128]. Similarly, age-related differences were observed in a desmopressin population PK model. These results contrast with data from human pediatric trials, probably because of a wider age range (neonates to 6 months) in the pig population. Moreover, a different model in pigs (i.e., two compartments, similarly to what was reported in adults) than in children (one compartment) was described. This, together with the limited number of children used in pediatric trials, led the authors to conclude that studies aimed at testing one vs. two-compartments models in children are necessary [129]. This is, in our opinion, good evidence that the pig can be superior than other model species in representing the human pediatric population, allowing for more accurate dosing in children. Also, results obtained in nonclinical studies with pigs should be combined with data from clinical trials and the data should be revisited when conflicting results are obtained. Despite the efforts to better characterize the neonatal and juvenile pig PK for different drugs, more research is necessary for the development of more accurate models.

Pig models have also potential as nonrodent species in the development of biopharmaceuticals, Europe being particularly interested in further exploration of the model [130]. So far, most of the data available relate to toxicology studies and nontraditional application routes, such as dermal or intra-ocular. Recently, the pig is gaining interest from a PK point of view: a variety of different molecules have been tested on pigs, including growth hormones, vaccines and several approaches of gene therapy. For example, the efficacy of growth hormone and analogues has been proved in pigs with compromised growth [131,132], although PK studies are lacking. The high similarities between the pig and human genome may make the pig a good model for hormonal therapies and thus, research in this field should be encouraged. In agreement with this, a recent study on somapacitan, a growth hormone derivative, showed similar PK and PD behaviors in minipigs, monkeys and hypophysectomized rats [133], the golden standard animal model for studying growth hormone pharmacology. Pigs have been more extensively used in the development of vaccines. A tuberculosis vaccine was tested in neonatal pigs and a similar immunological response by means of T cells and monocytes to that described in previous studies in infants was observed. This supports further development of the pig as a model with which to test the efficacy of pediatric vaccines [134], although the level of protection against *Mycobacterium tuberculosis* could not be determined in this study. Similarly, juvenile pigs (7-week-old) have been used for efficacy testing of a different (oral) administration route for Influenza A, with promising results [135]. The pig is a good model for influenza vaccine testing for obvious reasons: the same influenza viruses are endemic in pigs and humans and a similar clinical disease and pathogenesis have been reported [136], conferring the model an important target (i.e., similar molecular mechanisms participating in the pathogenesis of the disease) and predictive (i.e., response of the model to the pharmacological effects of treatments) validity [137]. Juvenile pigs were also used in the nonclinical evaluation of a new buccal form of Measles vaccine, with positive results [138].

A novel research topic within therapeutic research is genetic therapy, which includes a variety of approaches to treat disease by regulating, repairing, replacing, adding or deleting a genetic sequence, as defined by the EMA. Pigs have been used to test toxicity of a high dose of an adeno-associated vector expressing human survival motor neuron protein, together with NHPs. Both species showed neuron toxicity, which raised concerns regarding the potential side effects of this kind of therapy [139]. Some other works on gene therapy using neonatal and juvenile pigs use specific models of disease, and will therefore be discussed in Section 3.2.2. Our group aims to fully characterize a slightly different kind of gene therapy, consisting of the use of antisense oligonucleotides (ASOs). ASOs are a promising drug modality that, amongst other mechanisms, inhibit the translation of currently undruggable disease-causing proteins. ASOs are typically oligomers of 12–24 modified nucleic acids designed to target specific mRNA sequences [140]. Currently, there are no specific guidelines that regulate the authorization of ASO therapy candidates. For this reason, the nonclinical testing strategy of new chemical entities is followed for the safety assessment of ASOs [141]. Nonhuman primates have been a popular nonrodent choice because of their PK similarities and high genetic homology with humans. However, with the sequencing of the minipig genome [83,142], designing cross-reactive ASOs and evaluating pharmacology-related adverse effects became feasible in this porcine strain. The adult Göttingen Minipig has already been characterized to be a suitable alternative model for NHPs in the adult safety assessment of ASO drugs [143]. Target binding by ASOs was demonstrated and the toxicokinetic behavior in plasma, kidney and liver was similar compared to NHPs [143]. ASOs are well-absorbed in the circulation following subcutaneous administration. Transiently, they bind to plasma proteins (primarily albumin) across all species and this supports tissue distribution and hinders renal filtration [144,145]. Evaluating interactions of future ASO drug candidates with plasma proteins (as well as interactions with other competing drug substrates) in the juvenile minipig is essential since their plasma albumin levels may undergo maturation [63] and low plasma protein in younger animals can cause changes in drug distribution [97]. Nonetheless, as observed in different mammalian species, ASOs usually accumulate primarily in the kidney and liver, followed by a long elimination phase. Accumulation-related histopathological changes in these organs, causing nephrotoxicity and hepatotoxicity, are commonly observed in nonclinical testing of ASOs [146,147,148]. Aside from accumulation-related toxicities, nonspecific binding of ASOs to platelet-related proteins (i.e., platelet factor 4 and platelet collagen receptor VI) with corresponding platelet activation induces thrombocytopenia [149]. Similar effects have been observed in the adult minipig [143]. As fluctuations in platelet counts and coagulation parameters (prothrombin time, activated partial thromboplastin time) occur in the juvenile conventional pig and Göttingen Minipig [150,151], these factors may be of practical importance during pediatric safety testing of ASO drug candidates. Lastly, ASOs do not cross the intact blood-brain barrier (BBB) [152]. However, no data is available regarding the functional maturation of the BBB in the pig or the extent of access of ASOs into the developing brain of neonatal and juvenile pigs. The BBB undergoes functional postnatal modulation to provide a suitable microenvironment for the developing brain [153], and this may allow brain penetration of ASOs, resulting in neurotoxicity that is not relevant for humans. Therefore, BBB function in the neonatal and juvenile minipig is currently being investigated in our group in order to ultimately qualify the juvenile Göttingen Minipig as a pediatric safety testing model for ASOs.

Despite the numerous advantages of the pig over other models and the recommendations on selecting the best species based on scientific principles [29], statistics show a limited inclusion of the pig in PIPs during pediatric drug research. A still-developing characterization of the pig on the one hand and pharmaceutical companies being more used to working with traditional nonrodent species such as dogs and NHP on the other hand, may explain the limited use of pigs in pediatric drug development programs. However, efforts are continuously being made in our and other groups, to better characterize the physiological and pharmacological parameters of the developing pig. A recent review on vehicle systems and excipients tolerated by adult minipigs may also help investigators to more frequently consider the minipig as a nonclinical species [154], although this information is still lacking for neonatal and juvenile pigs. We believe that more training and facilitating the use of this species in pharmaceutical companies would also help to spread their adoption as nonclinical species in pediatric drug safety assessment. However, the pig occupies a privileged place when it comes to others aspects of welfare in the pediatric population, as will be discussed in the following sections.

#### 3.2.2. Examples of Pig Models in Condition-Specific Drug Development Research

Although normal, healthy pigs represent a good model in many cases, recently available genetic engineering techniques and the inherent characteristics of the species have given rise to the emergence of a wide variety of pig models representing active disease states. By either genetic, nutritional, surgical or medical manipulation, it is possible to develop tailored models used in biomedical, but also pharmaceutical research. In this section, we discuss some of the most relevant/common animal models used in pediatric drug development.

##### Neonatal Asphyxia and Therapeutic Hypothermia: Effects on Drug PK

Perinatal asphyxia (PA)-induced brain injury may be presented as hypoxic-ischemic encephalopathy (HIE) in the neonatal period, and may lead to permanent neurological impairment and long-term disability, such as cerebral palsy. The brain injury is secondary to the hypoxic ischemic event and the reoxygenation-reperfusion following resuscitation. An excess of nitric oxide is produced in the first hours after birth, which causes brain cell damage. In this regard, EMA approved a PIP using newborn pigs as a well-established model of PA [26,155,156,157] with the nitric oxide synthase inhibitor 2-iminobiotin. This drug was approved as an orphan drug as it reduces brain damage in the first 24 h after birth [158].

At present, therapeutic hypothermia (TH) is the only established treatment for moderate to severe HIE in term neonates [159]. This represents the standard treatment for neonates (≥36 weeks) with PA and HIE, since 2010 [160]. The benefits of hypothermia on survival and neurodevelopment outweigh the short-term adverse effects. These benefits are due to reduced metabolic rate and decreased neuronal apoptosis. Since both cooling and PA influence physiology, they are expected to alter PK and PD processes [26]. The effects and side effects of oxidative stress reducing/limiting medications may, however, be difficult to predict [159] and tools to support prediction of personalized dosing in this setting are lacking [161,162].

As the effects of PA and cooling on PK and PD cannot be assessed as separate variables in a clinical setting, animal models can provide useful information. Several protocols to induce perinatal asphyxia in pigs, are available, such as the creation of pneumothorax [163], halting mechanical ventilation [164] and, for cerebral ischemia, middle cerebral artery occlusion—MCAO and permanent internal carotid artery occlusion—ICAO [165]. Cheung et al. (2011) used surgically instrumented newborn pigs, involved in a nonsurvival surgical procedure, that allow the establishment of mechanical ventilation, vascular (arterial and central venous) access and the placement of catheters and flow probes for the continuously monitoring of intra-vascular pressure and blood flow across different arteries [166]. Severe hypoxemia is achieved by decreasing the inspired oxygen concentration to 10–15% and by increasing the concentration of inhaled nitrogen gas for 2 h, aiming for arterial oxygen saturations of 30–40%. This degree of hypoxemia will produce clinical asphyxia, with severe metabolic acidosis, systemic hypotension and cardiogenic shock with hypoperfusion to vital organs. The experiment includes 2 h of hypoxia, which results in PA comparable to clinical human situations in case of emergency cesarean section for fetal distress. The hypoxia is followed by reoxygenation with 100% oxygen for 0.5 h instead of 1 h and then 21% oxygen for 3.5 h. Optimal management of oxygen during neonatal resuscitation becomes particularly important because of the evidence that either insufficient or excessive oxygenation can be harmful to the newborn [167]. A previous study, using the neonatal pig model, provided morphological evidence that, after asphyxia, the ventilation with 100% oxygen is not superior to room air in most brain areas. Consequently, oxygen toxicity after asphyxia was demonstrated in the pig hippocampus and cerebellum, and not in the cerebral cortex or basal ganglia [164]. Relating clinical observations to post-mortem brain histology assessment in pigs could allow for a better interpretation of clinical observations, which could translate into a better fine-tuning and even the development of “personalized” reoxygenation protocols based on clinical changes.

Regarding drug therapy in these patients, analgesics (e.g., opioids) are commonly combined with sedatives (e.g., midazolam) in term babies with PA and are exposed to TH [168]. Drugs used in neonatal intensive care, such as midazolam, fentanyl, propofol, vecuronium, phenobarbital and propranolol, all have reduced clearance [169,170,171]. In this regard, evidence-based data on PK of different drugs used in neonates who underwent HT, as well as the knowledge gaps of the impact of PA and TH on ADME of these drugs in neonates, have been reviewed [26,168,172]. As an example, dexmedetomidine clearance was reduced almost tenfold compared with adult values in the newborn pig following hypoxic-ischemic brain injury and subsequent HT. Reduced clearance was related to cumulative effects of both hypothermia and exposure to hypoxia [169]. In our group, we are further investigating the role of HT and PA, separately and combined, on the PK of several other drugs that are used in the NICU.

In addition, the newborn pig is used as a model to study resuscitation practices. In fact, some recommendations of the International Liaison Committee on Resuscitation are solely based on newborn pig studies. Pasquin et al. compared different compression to ventilation ratios for resuscitation and found no better alternative than the recommended 3:1 ratio [173]. Solevag et al. showed a negative effect of using 100% oxygen during cardiopulmonary resuscitation compared to room air on myocardial function [174]. Furthermore, the newborn pig model was used to study the effect of epinephrine and fluid boluses [175].

##### The Special Case of the Neonatal Preterm Pig

The preterm pig deserves special mention due to the significance of the model in the fields of neonatal respiratory and intestinal tract conditions in preterms. These two organ systems are involved in most of the cases of neonatal hospitalizations: firstly, respiratory failure is one of the main causes of referral to the neonatal intensive care unit (31.0%). Secondly, out of those premature babies who survive the first days, the onset of intestinal diseases such as NEC again challenges their survival [176]. Both conditions are also present in preterm pigs obtained by cesarean section at about 90–95% gestation. Moreover, the relevance of preterm pigs as models in the area of neurodevelopment, related to (mostly nutritional) strategies to stimulate brain development and cognition is manifest: Supplementation with cholesterol, sialic acid compounds (including lactoferrin), phospholipids and antioxidant present in (human) milk, iron, choline and betaine affected brain development, behavior, learning and the expression of genes involved in these processes in pigs (reviewed in [28]).

1. Respiratory Pathologies and Lung Development

The newborn pig is a well-known model for studies in respiratory diseases. Preterm born pigs in a spontaneous nontreated infant respiratory distress syndrome (IRDS) study showed consolidated lungs, immature alveolar architecture and minimal surfactant protein-B expression (SP-B) at GDA 98 and GDA 100 (term at 114 days). From GDA 102 thin-walled alveoli were present lined with pneumocytes expressing SP-B. Before GDA 98 the development of pig lungs is in the canalicular phase comparable with human infants before 24 weeks of gestation. Between GDA 98 and GDA 104 the lungs develop in the saccular phase, alveolar ducts are formed and lined with type II cells, comparable with 24–34 weeks of human gestation. Preterm pigs showed strong resemblance to human preterm infants in the pattern of lung development and the presentation of IRDS, clinically and histopathologically [177]. Eiby et al. described the physiological and anthropometric characteristics of the (pre)term pig from GDA 91 till term [178]. They showed that pigs can be resuscitated and kept alive from GDA 96 using standard neonatal intensive treatment including artificial ventilation and surfactant administration. Also, maternal corticosteroid administration was shown to improve respiratory and hemodynamic stabilization in preterm pigs [178]. Preterm born pigs were studied to compare ventilator strategies from GDA 97 as a model for neonatal RDS [179,180]. Other studies used term born pigs in an acute newborn lung injury model by repeated bronchoalveolar lavage, meconium instillation, injurious ventilation and intratracheal lipopolysaccharides instillation. Characteristics of lung function, immune response, hemodynamic during ventilation and renal function were described. The newborn pig has many similarities to the human newborn including lung development, the presence of lung macrophages, NO production, the response to lipopolysaccharides and the hypervariable region of toll like receptor 4 [54,68,181]. The latter neonatal acute lung injury model was also used for nonclinical studies of high frequency oscillatory ventilation and (partial) liquid ventilation using perfluorocarbons [182,183].

In addition, the pig is used as a model for cystic fibrosis. Pigs were generated with a typical delta F508 deletion encoding for the CF transmembrane conductance regulator (CFTR). These pigs show problems in eradicating bacteria from the airways and show lung disease with inflammation, remodeling, mucus accumulation and infection [184].

2. Digestive Alterations

The preterm pig is probably the most recognized model when it comes to prenatal nutrition and gastroenterology. Besides research on development of dietary interventions aimed at improving overall health, development and growth of preterm infants, the preterm pig is also used as a model of two diseases: NEC and short bowel syndrome (SBS). These two intestinal diseases predominate within the preterm neonatal population and are interconnected.

Necrotizing enterocolitis is a devastating and potentially lethal gastrointestinal disease in preterm neonates. So far, the best measures available to control its consequences aim at accelerating the maturation of the GI tract, improving bacterial colonization and providing optimal nutrition consisting of human milk, since formula feeding is one of the risk factors [176]. The preterm pig model of NEC following enteral feeding with infant formula was first described by Sangild et al. in 2006 [185] and demonstrates a similar pathogenesis in both species. Since then, numerous nutritional strategies have been tested in preterm pigs, including delayed initiation of enteral feeding [186] and the use of milk and colostrum (from different species including human, cow and pig) rather than formula [185,187,188]. Moreover, the better response to colostrum than milk is related to the abundance of growth factors and immune related proteins in the former. In this line, glucagon-like peptide 2 (GLP-2) has been shown to induce marked gut mucosal growth and blood flow in neonatal pigs and to delay, although not prevent, the onset of NEC in preterm pigs [189]. Other components, such as galacto-oligosaccharides, bovine lactoferrin and milk fat globule membrane-10 supplemented to the newborn pig as well as colostrum/milk but not formula, improved intestinal development, in agreement with studies in human babies (reviewed in [176]). Moreover, fecal filtrate transplantation [190], lactoferrin and probiotics, [191], as well as broad-spectrum antibiotics [192] proved to be a good strategy to prevent NEC by modifying GI microbial communities and reducing intestinal inflammation, although antibiotics altered pig metabolism [193]. Moreover, it has been reported that antibiotics work against NEC onset when provided enterally but not parenterally [194]. To avoid this and other side effects of antimicrobial therapy and live probiotics in immature babies, a potential alternative consisting of the treatment with CpG-containing bacterial DNA is effective against experimental NEC in pigs. The preterm pig is therefore a valuable model for human NEC and should be (as has been) used to test novel management, nutritional and pharmaceutical strategies to improve the outcome of babies suffering from this condition.

Short bowel syndrome is a condition where either anatomical or functional loss of a significant length of the small intestine result in nutrients malabsorption. Although patients from basically all ages can suffer from SBS, the incidence is 100 times higher in premature babies [195], due to the higher incidence of NEC. Thus, the preterm pig has been extensively used as a model in biomedical research as well as in the development of therapies, including dietary [196], pharmacological and even surgical [197] approaches. Among the drugs and biopharmaceuticals tested in pigs to ameliorate SBS symptoms and to improve intestinal adaptation and function, human recombinant GH [198] has been tested with limited efficacy to improve clinical outcomes and intestinal adaptation and function. The supplementation of enteral nutrition with bioactive compounds such as butyrate [199] or the use of colostrum, with high concentration in IGF-1 [200], have also been tested in SBS pigs. However, none of these proved to be effective. In spite of the limited effects of these strategies, success was attained with the use of GLP-2 and its analogues, positioning these molecules as the most relevant ones from a clinical standpoint. Human GLP-2 administered to an SBS pig model, alone or in combination with epidermal growth factor (EGF) [201], improved intestinal morphology and decreased the time on parenteral nutrition [202]. EGF and GLP-2 showed a synergistic positive effect on intestinal morphology but differences in nutrients absorption were observed [201]. Since GLP-2 is a gut growth factor with a very short half-life, the efficacy of different long-half-life analogues has been tested in recent years. In 2014, the analogue teduglutide, used in the treatment of SBS in adults [203] but not tested in pediatric population at that time, showed a positive trophic effect in pigs but limited improvement on functional endpoints such as activity of digestive enzymes or nutrient absorption [204]. Of interest, this drug is currently approved for adults and children aged 1 and above. More recently, the administration of a novel, long-lasting GLP-2 analogue (apraglutide) to neonatal pigs led to better nutrients (fat and energy) absorption [205]. In conclusion, studies in SBS pig models allow assessing the safety and efficacy of GLP-2 and its analogues, which could be translated into pediatric patients.

Another common digestive alteration where the pig (in this case, mostly term-early-weaned pigs) is used is irritable bowel syndrome (IBS) (reviewed in [206]). In humans, it has been suggested that such gastrointestinal dysfunction can occur at a young age and is induced by stress [207]. The stress response to weaning in the pig is remarkably similar to what is seen in humans: it is characterized by changes in immunological parameters, distorted redox balances, increased intestinal permeability and diarrhea [208]. Another role in the stress-related responses, is to be played by the (intrinsic) enteric nervous system. The different enteric neuron types, their morphology, electrophysiology, projections and connectivity are more similar in the pig than in other species, when compared to humans [209]. Moreover, the fact that possible risk factors such as birth weight, the level of maturity, the diet where shown to affect different important players in IBS (immune system, redox balance, enteric nervous system) [210,211,212,213,214,215,216] adds to the relevance of the (young) pig for exploring the mechanism and treatments of these neurogastroenterological disorders.

##### Other Relevant Models from a Clinical Perspective

Neonatal and juvenile pigs also serve as models for many other conditions, with a smaller impact in the pharmaceutical industry, either due to the presence of other equivalent models or to the lesser amount of research performed in those areas. Some of the most important ones will be briefly discussed in this section. General and more commonly used models are discussed in detail, while disease-specific models are only briefly described. We refer the reader to the original papers for a better understanding of the model.

Intrauterine growth restricted (IUGR) pigs share numerous characteristics with IUGR babies and are thus extensively used as a model for this condition, in which the neonate is born with a smaller size than determined by their genetic potential. Most of these alterations are due to the improper development of several organs and systems. Although a majority of the research performed in IUGR pigs focuses on the study of the physiology of IUGR pigs and its translation into humans (as reviewed in [217]), the model is also used in the search of therapeutics. Supplements and nutraceuticals have been administered to pregnant sows to determine their efficacy in reducing the incidence [218] and the severity of IUGR [219,220], although interventions to the pig itself are more frequent. In this regard, dietary supplementation with nucleotides improved nutrients utilization, intestinal function and immunity in a pig model of IUGR [221]. Beneficial effects on the redox status and mitochondrial function with translation potential have also been observed after the administration of *N*-acetylcysteine [222], resveratrol [223] and curcumin [224] to IUGR neonatal pigs. Interesting therapeutic perspectives exist also for insulin, which demonstrated to have the ability to partially reverse the IUGR phenotype in a pig model [225]. Similar to what has been described for the preterm pig model, the IUGR pig model is also used in neurodevelopment and neurotoxicity studies, although to a lesser extent. For example, the toxic effects of isoflurane and nitrous oxide anesthesia, as measured by neuro-apoptosis, were reported to be higher in IUGR than normal pigs, which should be considered when anesthetizing infants born with IUGR [226]. Moreover, the brain damage and inflammation observed in babies and in the IUGR pig model could be reduced by administration of ibuprofen for three days [227]. Considering the recent data on ibuprofen PK in neonatal pigs, the implementation of ibuprofen for this indication (different from the labelled indication to induce closure of the patent ductus arteriosus) in the treatment of IUGR babies could soon be a fact, as the drug is registered for use in neonates and the enantiomer specific PK in small for gestational age setting (3.1-fold higher *S*-ibuprofen clearance in small for gestational age cases) were recently reported [228].

One of the biggest concerns regarding IUGR neonates is the higher risk of metabolic diseases and obesity observed in future life [229]. This has led to the search for optimal nutritional management of these babies and kids, as reflected in preliminary studies addressing the effects of nutrients or energy restrictions on neonatal [230] and weaning pig models [231,232]. This is also related to the fact that pediatric obesity and nonalcoholic fatty liver disease (NAFLD) are on the rise in developed countries. In recent years, juvenile Iberian and Ossabaw pigs are put forward as large animal models to study both disorders which can be elicited via challenging these pigs with a Western diet [233,234,235]. Although rodent models are typically used to study adult obesity and NALFD, they have limited use in studying the mechanisms of these diseases in infants and children. Moreover, mice do not develop the severe liver inflammation and fibrosis [236], whereas the dietary challenged pigs develop these hallmarks. Recent reports have pointed to the microbiota [233] and dietary fat sources [234] to play an important role. More recent studies have described in more detail the biochemical and signaling pathways that go astray and lead to NAFLD in the juvenile pig [237] and provide cues on possible therapeutic interventions.

Neonatal/juvenile porcine models have also been developed to mimic inflammatory processes in humans. For example, neonatal pigs were used to develop a model of chronic peritonitis by ligating and puncturing the cecum, with the goal of testing the effects of insulin on muscle protein metabolism [238]. More extreme cases of inflammation may lead to endotoxic shock, which can be reproduced in pediatric pig models by the administration of lipopolysaccharides [239,240] or *E. Coli* endotoxin [241]. These models have been further used to test efficacy of vasoactive molecules -dobutamine and vasopressin- on blood flow [241] and allowed the identification of side effects related to the use of 1-methyltryptophan, which should be considered during its therapeutic application [239].

Finally, another area of research—previously mentioned in this review—where pediatric pig models are used is gene therapy. They mostly account for genetic disorders, such as Duchenne muscular dystrophy [242], cystic fibrosis [243,244] or hereditary tyrosinemia type 1 [245], although models for nonischemic heart failure have also been developed [246]. The aforementioned pig models have been used to test toxicity [245,246], biodistribution [246] and efficacy [244,245,246] of different vectors used in gene therapy.

## 4. Pitfalls, Limitations and Unexplored Areas of the Model

### 4.1. Imbalance between Disease Model and Drug Development

The developmental, physiological and anatomical similarities between pigs and humans, as well as the plasticity of the pig in mimicking pediatric conditions, is astonishing. These features have conferred great recognition and popularity on the pig within the scientific community. Nevertheless, the use of the species is often limited to fundamental translational research among public institutions. This kind of experiments usually generate knowledge on molecular mechanisms underlying clinical observations and can be considered a first step toward the identification of pharmaceutical targets and thus, drug development. However, when we advance in the pediatric drug development process, the number of efficacy studies using pigs decreases. The development of new models for specific conditions and the availability of transgenic pigs is nonetheless changing this trend, as illustrated in Section 3.2.2 of the present review. Moving further into the drug development process, the pig is rarely used among pharmaceutical companies as a nonclinical species [247,248], despite the extensive literature that supports the equivalence and even superiority of the pig over the dog as model species in toxicity studies both at adult [247,248] and juvenile stages [249,250]. This reveals an imbalance between public and private institutions in the use of the pig as model and disparity in the role of the pig in fundamental and pharmaceutical research, the latter being still largely unexplored. As briefly discussed earlier in this review, we believe that the incomplete characterization of the species, the limited control data and standardized phenotyping protocols and the lack of experience in pharmaceutical companies are key to this imbalance.

Continuous efforts are being made to better characterize the (juvenile) pig in the fields of ophthalmology [251,252], neurodevelopment [28], immunology [28], and drug absorption, metabolism and toxicity [35,43,45,253,254,255,256], although there is still evidence supporting the need for more background information in pigs [253]. Moreover, differences in between breeds exist [257] and, since fully characterizing all the pig breeds available is a long-term task, most efforts concentrate in studying the most used pig breed in pharmacological studies in Europe, the Göttingen Minipig [35,43,44,45,258,259,260,261,262,263,264,265,266,267,268,269,270,271,272,273,274]. Inter-breed differences are magnified due to the lack of standardization regarding food and feeding patterns, age and sex of pigs used in research [275]. To improve the characterization of the pig, phenotyping techniques and assessment protocols are also needed to enable progress. For example, the pig is less used than the rat in neurodevelopmental studies due to the lack of tests to assess learning and memory. Fortunately, test methods are being developed [29]. In this line, guides for standardization of sampling protocols [276,277], welfare assessment [278] and description of protocols to apply human diagnostic tools to pigs (reviewed by [28]) are available [30] and should be followed in order to decrease variability and obtain more and better control data. The ongoing development [279] of universal protocols and good laboratory practice manuals for handling and sampling pigs in translational research will probably increase the acceptance of these guides by the scientific community. The limited acceptance of the pediatric pig model by pharmaceutical companies may be driven by the fact that pharmaceutical companies scarcely use adult pigs (but rather dogs, as discussed in Section 3.2.1) in repeated dose toxicity studies, since selected species are only required to share similar molecular targets and pathways with humans. Although this trend is already changing and pigs are increasingly being used as an alternative to the dog or NHP [29,42], we believe that stricter requirements for selection of nonclinical species regarding similar pathophysiology would lead to more accurate predictions and broader use of pigs. Defining more detailed species selection criteria in the current regulatory guidelines for adult repeated dose toxicity studies, e.g., which species to be considered, would also persuade pharmaceutical companies to take into account the Göttingen Minipig in their nonclinical testing strategy, including pediatrics. In this respect, the ICH S11 guidelines [36] clearly state that the same species as used in adult repeated dose toxicity studies should initially be considered as the species for a juvenile animal study, preferably a rodent. This explains that the rat is the most commonly used pediatric safety testing model and the very limited use of the Göttingen Minipig in PIPs, as several pharmaceutical companies currently do not consider the Göttingen Minipig for their adult nonclinical program.

### 4.2. Differences in Organ Maturation

As in any other animal model, differences between neonatal and juvenile pigs and humans exist, and should be considered in the species selection process. Pigs are born smaller than humans (around 0.5 kg for a Göttingen Minipig and 1.5 kg for a domestic pig) but they experience a more rapid growth. Göttingen Minipig show a linear growth up until 18 months, when they reach about 40 kg BW. Domestic pigs on the other hand present more of an exponential growth curve until 4 months of age. Some anatomical physiological and maturation differences between the two species have been already discussed in Section 2.1 of the present review. For clarity, we will approach this section in a similar way to that used in the aforementioned section (i.e., by system).

As previously mentioned, the digestive system is the one where more similarities are observed, especially from a physiological point of view. There are, however, some inconsistencies between the porcine and the human GIT. The prominent cecum, the lack of an appendix, the larger colon, and differences in gut-associated lymphoid tissue (GALT) [280,281] should be considered. In addition, some differences are observed at the level of the ultrastructure: human neonates show broad leaf-like intestinal villi that evolve into longer, finger like villi in adulthood [282]. In contrast, pigs present finger-like villi during the preweaning period and shorter and wider villi (leaf-like) after weaning [283]. Regarding the GALT, anatomical differences have been identified in the development, distribution, cellular composition and number of Peyer’s patches between pigs and humans [284]. In addition, there is controversy of the presence or absence of Paneth cells in the pig [285,286,287]. The functional significance of these morphological differences remains unclear. From a physiological standpoint, perhaps the most important difference between humans and pigs relates to the faster change of gastric pH toward adult levels observed in pigs when compared to human (see Section 2.1). Finally, although the microbiome is similar at the phylum level, there are differences at the genera level and notably Bifidobacteria are only present in low amounts in the pig [288,289]. The human and porcine pancreas show numerous anatomic differences and a different ontogeny in pancreatic enzymes [40] but they are comparable when it comes to the endocrine function. In fact, pig pancreas islet xenotransplantation into NHPS is already a reality and the potential pig-to-human transplantation of islets is being explored [290]. Moreover, porcine models of diabetes are not uncommon [291,292], which illustrates the similarities in pancreas physiology between pigs and humans. As aforementioned, the juvenile human liver is relatively heavier when compared to the porcine liver. Also, differences in the ontogeny of drug metabolizing enzymes have been reviewed in Section 2.2. Regarding the cardiovascular system, anatomical differences between the neonatal and adult human and pig heart have been reviewed elsewhere [46]. However, the growth of the porcine heart and cardiovascular system from birth to 4 months of age is analogous to the growth of the same system in humans into the mid-teen [29]. Also, a characterization of the juvenile minipig has been performed, which highlighted some differences, for example in the gastroepiploic artery, between species [260].

Regarding the CNS, many similarities in anatomy and development are reported between the two species, including maturation of cerebral white mater [293], composition and electrical activity [294]. Perhaps one of the most remarkable differences is the presence of a rete mirabile in the pig [40] participating in the irrigation of the brain. Moreover, differences between pig strains have been reported, e.g., a significant development of neurons and glial cells occurs postnatally in the Göttingen Minipig, whereas the adult number of neurons is established at birth in the domestic pig [294], and therefore, strain differences must be considered in neurodevelopment studies. The porcine and human kidney differ in the maturation of nephrons, which is faster in humans, as discussed in Section 2.1. Moreover, a lower GFR (mL/min/m^2^) is reported in humans (around 55 to 80% the values in pigs), but the maturation of the GFR seems to be comparable [66]. It is also important to notice that a high prevalence of infiltration of inflammatory cells has been reported in different strains of minipigs [295], which should also be taken into consideration when assessing nephrotoxicity.

Perhaps one of the most relevant differences in the reproductive system from a translational point of view concerns the structure of the placenta (i.e., epitheliochorial placentation in the pig vs. the hemochorial human placenta). This difference, despite being a limitation in the field of reproductive toxicology [130], can be an advantage for studies on immune system development as previously mentioned. Moreover, it determines the transmission route of immunoglobulins (trans-placental in human and via colostrum in pigs), which should be considered in perinatal or neonatal studies. Pigs are also more precocious in regards to the onset of puberty, 3 vs. 17% of the pig and human lifespan, respectively [296]. Other than that, differences in the uterine structure, which consist of two horns in the case of the female pig, and in the length of the cycle (3 vs. 4 weeks in women) and origin of the luteolyzing hormone (uterus vs. ovary in women) have also been described. Moreover, the porcine vaginal flora is not dominated by lactobacilli, as opposed to the human flora and the pH is slightly higher in adult sows than women [297]. Regarding the male reproductive tract, the porcine penis contains less erectile tissue when compared to human and is corkscrew-shaped, which hinders catheterization of the bladder. Moreover, hypoplasia of the seminiferous tubules has been observed in minipigs during toxicological studies [29]. Other background alterations more commonly found in minipigs than human relate to testicular hypoplasia/degeneration [295].

Regarding the respiratory system, the lungs of pigs and humans are quite similar, although there are some minor differences. The porcine trachea is longer than the human and more cartilaginous. Furthermore, the porcine right lung consists of four lobes compared to three in humans and between alveoli human lungs have more interalveolar pores facilitating collateral ventilation. They show the same number of bronchial generations although the porcine bronchial tree shows a monopodial branching pattern with several side branches from one parent bronchus, whereas the human bronchial tree shows a bipodial branching pattern [70]. As already mentioned before, newborn pigs are more precocious compared to newborn humans. With respect to the development of the respiratory system, pig lungs show the canalicular phase before gestation d98 (85% of gestation) compared to 24 weeks of gestation (60%) in human fetuses. The next saccular phase ends around 104 (91%) days of gestation in the pig and 34 weeks (85%) of gestation in human fetuses [177]. After birth, porcine alveoli multiply and lung volume increases much faster than in humans [70].

In line with differences in placentation, the most remarkable difference in regards to the immune system between pigs and humans is the lack of passive immunity at birth in the former. Other differences, less significant from a translational point of view, include the thymus is located in the neck in the pig vs. in the mediastinal space in humans and its cellular and morphological maturation is slower in the minipig. Similarly, human unlike porcine spleen is already mature at birth [298]. Despite these differences, the development of the immune system from a functional standpoint is quite similar and, as mentioned in Section 2.1, the pig represents a good model in immune-related research.

The pig, as a precocial species, presents well-developed musculoskeletal and neuromuscular systems when compared to humans, as discussed in Section 2.1. Moreover, the bone mass in pigs is greater than in humans and, while pig’s epiphyseal closure occurs well into adulthood (2 to 4 years of age, depending on the strain) [29], this happens at the end of the pubertal stage in humans [299]. Although the musculoskeletal system was one of the aspects in which pigs have been traditionally considered as mediocre or bad models, a recent interest on the translational value of the species in the field tissue engineering and regenerative medicine has emerged, as reviewed by Cone et al. [300]. Juvenile models are often included in these studies. Finally, anatomical differences, including the lack of eccrine sweat glands in the pig have been described among the two species have been reviewed elsewhere [40]. However, since the physiological similarities are remarkable, the pig is still considered a good model in topical administration studies.

### 4.3. Limitations of the Current Pig Proteome

Although convenient under laboratory conditions and efficient to simulate and model diseases, model organisms often fail to reflect human physiology and pathology. Due to the necessity of characterizing species on the molecular biology and genetic level [42], all model organisms are fully sequenced and well annotated but nonmodel species are not. Although pigs are not considered as classical model organisms, they are an excellent tool to study human physiology and pathology. They are genetically more similar to humans than mice. The first full pig reference genome sequence assembly was published in 2005. The pig genome is ~2500 Mb in size and contains 18 autosomal and two sex chromosomes with similar sizes to humans [301]. Proteomics studies have covered both physiological and biomedical application studies of pig to a much greater extent than for other farm animals [302,303]. Pig breeders often can provide access to a wide range of genetically well-defined breeds and families. Animals selected for breeding are genotyped routinely, providing a detailed insight on the genetic variations in thousands of genes across the entire genome [304]. Although pigs are more and more used as models, proteomics literature by no means yet reflects the broad range of existing porcine medical models research [305]. However, times are changing. Advances in porcine genomics and proteomics will provide the breakthrough for fully exploiting the use of porcine models for biomedical research though -omics technologies. The sequencing of genomes of different strains, the calling of genetic variants, the building of porcine peptide and mRNA mapping data repositories are a great leap forward for the usage of pigs as models. Also, the genome of the mini pig has been sequenced [83]. Although for pig the publicly available proteome data is still lagging behind in the proteome data repositories (Pride (http://www.ebi.ac.uk/pride), PeptideAtlas (http://www.peptideatlas.org/repository), GPMDB (Global proteome Machine Database) (http://gpmdb.thegpm.org/) and the NCBI-peptidome (http://www.ncbi.nlm.nih.gov/projects/peptidome); all databases include increasing amounts of data [306] filling the gap. The Pig Peptide Atlas currently contains 320 experiments and covers data from more than from 15 major porcine tissues. Functional annotation is still a challenge in nonmodel or new model organisms and so also in pig. Uniprot currently contains 3555 protein accessions in the well annotated database Swissprot while 178,195 poorly annotated accessions are present in Trembl (November 2021). As a comparison for mouse, 17,489 or almost the complete proteome is listed in swissprot (November 2021). So, incomplete GO annotations and mapped pathways still present challenges. This can be circumvented by using a homology-driven approach in which many annotations are inferred based on bioinformatic analyses. Another challenge is that only a few pig strains are fully sequenced, leading to failure to identify strain specific alleles. A way to circumvent this is performing an error tolerant search or de novo sequencing for protein identification [307] or by coupling proteomics to combining RNA-Seq [308,309,310,311,312,313,314,315].

### 4.4. Practical/Logistical Disadvantages

From a practical point of view, the pig presents some disadvantages when compared with the dog or other nonclinical species. Even the smallest breed commonly used in research (Göttingen Minipig) is heavier than Beagle dogs at any stage of development. The former born at around 0.5 kg bodyweight, and reaches 9 kg at 4 months of age, i.e., almost twice as heavy as a dog. This may represent an advantage when it comes to sampling at very early stages (even conventional pigs, born at around 1–2 kg BW, are used in short studies in very young animal when size can be advantageous) but tends to be more problematic when repeated doses of drugs are necessary, especially regarding compound consumption and availability. The field of gene therapy is nevertheless an exception, since limited number of lower doses are required. Financial limitations may also constraint the widespread use of the highly relevant preterm pig model, due to the need for a sow surgical facility, a pig intensive care unit and clinically trained personnel to take care of these pigs [316].

Blood sampling and IV administration of drugs may be more challenging than in dogs, since peripheral veins are not easily accessible. Thus, the implantation of IV catheters for repeated dosing is often required, although the jugular and cranial cava vein can be readily used for blood sampling by experienced personnel [30]. We have previously mentioned the importance (and the still common lack) of experience when working with minipigs, as with any other species. As the use of minipigs is increasing, personnel will become more acquainted and thus, more competent and confident with the species. There are nevertheless educational and training programs available [317,318] for new or less experienced companies wanting to work with minipigs.

Pigs are foragers and very curious animals; therefore, environmental enrichment, a legal obligation of the animal facility or lab, needs to be sufficient to satisfy these necessities and reduce stress levels. Moreover, blood pressure changes have been reported in stressed pigs. Finally, regulatory restrictions preventing the transport of minipigs for the prevention of the spread of contagious diseases among livestock may complicate the acquisition of (mini)pigs from breeding centers [319].

## 5. Conclusions and Expert Opinion

The physiology and development of several organ systems and conditions associated to preterm birth are very similar in pigs and humans. Despite this fact, the use of neonatal and juvenile Göttingen Minipigs, the reference breed in pharmaceutical industry, in pediatric drug development programs is still very limited. One of the main reasons for this is the fact that pediatric regulatory guidelines recommend using the same (preferably rodent) species and strain in juvenile animal studies as in adult repeated dose toxicity studies. As regulatory guidelines for adult repeated dose toxicity studies currently do not define species selection in a detailed manner, there is no consistent approach and pharmaceutical companies often base their decision for species selection on experience and data with rats and dogs over the years. As such, the Göttingen Minipig is not considered in their species selection process. In our opinion, including more detailed species selection criteria in the regulatory guidelines for adult repeated dose toxicity studies would increase the use of the Göttingen Minipig in drug safety testing for indications in the adult and pediatric population. For pediatric-only indications, especially in the youngest age groups in which extensive clinical and nonclinical studies in adults are, in general, not performed, pharmaceutical companies should consider by default the neonatal and juvenile Göttingen Minipig for their nonclinical program, as it often represents a better translational model than rat and dog pups. We anticipate that current efforts to fully characterize the model, including ADME processes, and the development of juvenile pig PBPK models will promote the use of the juvenile pig model in pediatric drug safety studies.

## Data Availability

No new data were created or analyzed in this study. Data sharing is not applicable to this article.
